# Identification of Quorum Sensing Activators and Inhibitors in The Marine Sponge *Sarcotragus spinosulus*

**DOI:** 10.3390/md18020127

**Published:** 2020-02-20

**Authors:** Kumar Saurav, Nicola Borbone, Ilia Burgsdorf, Roberta Teta, Alessia Caso, Rinat Bar-Shalom, Germana Esposito, Maya Britstein, Laura Steindler, Valeria Costantino

**Affiliations:** 1 Department of Marine Biology, Leon H. Charney School of Marine Sciences, University of Haifa, Mt. Carmel 31905, Haifa, Israel; sauravverma17@gmail.com (K.S.); burgsdorf84@gmail.com (I.B.); rbar-shal@univ.haifa.ac.il (R.B.-S.); mayabritstein@gmail.com (M.B.); lsteindler@univ.haifa.ac.il (L.S.); 2 The Blue Chemistry Lab, Dipartimento di Farmacia, Università degli Studi di Napoli Federico II, Via D. Montesano 49, 80131, Napoli, Italy; nicola.borbone@unina.it (N.B.); roberta.teta@unina.it (R.T.); alessia.caso@unina.it (A.C.); germana.esposito@unina.it (G.E.); 3 Laboratory of Algal Biotechnology-Centre Algatech, Institute of Microbiology of the Czech Academy of Sciences, Opatovickýmlýn, Novohradská 237, 379 81 Třeboň, Czech Republic

**Keywords:** sponge, quorum sensing, quorum sensing inhibition, *N*-acyl homoserine lactone, *Sarcotragus spinosulus*, 3-bromo-4-methoxyphenethylamine, 5,6-dibromo-*N*,*N*-dimethyltryptamine

## Abstract

Marine sponges, a well-documented prolific source of natural products, harbor highly diverse microbial communities. Their extracts were previously shown to contain quorum sensing (QS) signal molecules of the *N*-acyl homoserine lactone (AHL) type, known to orchestrate bacterial gene regulation. Some bacteria and eukaryotic organisms are known to produce molecules that can interfere with QS signaling, thus affecting microbial genetic regulation and function. In the present study, we established the production of both QS signal molecules as well as QS inhibitory (QSI) molecules in the sponge species *Sarcotragus spinosulus*. A total of eighteen saturated acyl chain AHLs were identified along with six unsaturated acyl chain AHLs. Bioassay-guided purification led to the isolation of two brominated metabolites with QSI activity. The structures of these compounds were elucidated by comparative spectral analysis of ^1^HNMR and HR-MS data and were identified as 3-bromo-4-methoxyphenethylamine (**1**) and 5,6-dibromo-*N*,*N*-dimethyltryptamine (**2**). The QSI activity of compounds **1** and **2** was evaluated using reporter gene assays for long- and short-chain AHL signals (*Escherichia coli* pSB1075 and *E. coli* pSB401, respectively). QSI activity was further confirmed by measuring dose-dependent inhibition of proteolytic activity and pyocyanin production in *Pseudomonas aeruginosa* PAO1. The obtained results show the coexistence of QS and QSI in *S. spinosulus*, a complex signal network that may mediate the orchestrated function of the microbiome within the sponge holobiont.

## 1. Introduction

Overuse of antibiotics is one of the factors involved in the emergence of drug-resistant pathogens. The discovery of alternative novel strategy to tackle these infections is required to solve this emergent problem. The understanding of how intercellular microbial communication is involved in bacterial pathogenesis has revealed potential for alternative strategies to treat bacteria-mediated diseases [1,2]. Cell–cell communication, quorum sensing (QS), is a cell-density dependent phenomenon that triggers the genetic regulation, coordinating the physiologies of the different cell types contributing directly to pathogenesis through the synchronized production of virulence determinants, such as toxins and proteases [3,4]. It has been theorized that, if the signal communication was blocked by different inhibitory mechanisms (quorum sensing inhibition, QSI) including enzymatic inactivation of the signal molecule [5,6], inhibition of signal biosynthesis [7], and inhibition of signal detection [8,9], bacteria would lose their ability to form organized community structures that confers antibiotic resistance [10].

Sponges (phylum Porifera) are an important component of aquatic benthic communities. Their arsenal of chemicals has been investigated in terms of chemical ecology (e.g., [11]), drug discovery [12], and for biotechnological purpose (e.g., [13]). Growing evidence suggests that microbial symbionts are the main producer of several documented sponge-derived bioactive compounds rather than the host itself alone [14,15,16,17]. Within the densely colonized sponge, there is an ample opportunity for intraspecies, interspecies, and interkingdom chemical signaling [18,19,20]. QS was shown to be essential for the successful establishment of symbiotic and pathogenic relationships with eukaryotic hosts [21]. The presence of *N*-acyl homoserine lactones (AHLs), QS molecules mediators in the sponge extracts, sponge- bacterial isolates, and a metagenomics-derived genome of a sponge symbiont (e.g., [19,20,22,23,24]) are well documented. On the contrary, few studies have reported the presence of QSI compounds in the sponges and their isolates. The concept that such molecules could provide alternatives for antimicrobials [25,26,27,28,29] has been recently discussed. While AHLs have a common structure, QSI compounds are structurally different: ranging from AHL-like molecules [30,31] to cyclic peptides [32,33,34], alkaloids [27,35], lactones [36], and diterpenes [37,38,39]. In our recent work, a novel lactone, named plakofuranolactone, which showed a strong QSI activity at sub-micromolar concentration, has been discovered from the extract of the Indonesian sponge *Plakortis* cf. *lita* [36].

Recently, we showed that some sponge species have constant presence of AHLs, while other species show high variability both in presence and type of AHLs. This variability was observed in the case of *Sarcotragus* sp., where 3 out of 18 specimens tested harbored AHLs and, based on chromatographic analysis, the AHL profiles differed among these specimens [24]. Such variability does not surprise being easily explained by differences in microbial communities among different specimens of the same species.

In this paper, we report data showing the presence of AHLs as well as QSI molecules in the sponge species *Sarcotargus spinosulus*; two brominated metabolites: 3-bromo-4-methoxyphenethylamine (**1**) and 5,6-dibromo-*N*,*N*-dimethyltryptamine (**2**), possessing QSI activity, were identified by bioassay-guided purification.

Moreover, in this study, we propose the cell-separation and extract fractionation as a useful tool to enrich the titer of the minor constituents. This methodology leads to the identification of additional AHLs, often missed in direct analysis of the extract.

## 2. Results and Discussion

### 2.1. Taxonomic Identification of Sponge

**Figure 1:**
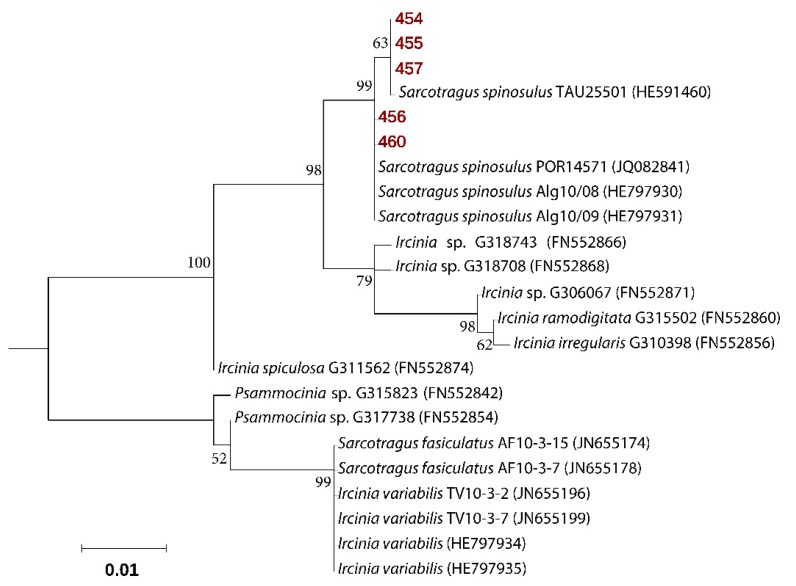
Molecular phylogenetic analysis based on cytochrome oxidase gene, subunit 1 sequences. The Maximum Likelihood tree is shown, with sequences repossess in this study highlighted in bold and red. Bootstrap values derive from 1000 replications and are shown at branch nodes. Values above 50% are shown.

Five samples of *S. spinosulus* were collected along the Mediterranean coast of Sdot Yam, Israel, during summer 2016. Sponge specimens used in this study (voucher no: 454, 455, 456, 457, and 460) displayed a high degree of intraspecific mitochondrial cytochrome oxidase subunit I (COX1) gene conservation with 99%–100% identity (E-value = 0.0) to the sequence published for *Sarcotragus spinosulus* (accession number HE591460). Maximum likelihood phylogenies also showed clustering of the sequences from five specimens, within the representatives of the *S. spinosulus* species (Figure 1). *S. spinosulus* is a massive southern species recorded in the Atlantic coasts and throughout the Mediterranean Sea.

### 2.2. Identification of AHLs in Sarcotragus spinosulus Crude Extracts

Out of the five sponge specimens, four gave a response to two AHL-biosensor tests, i.e., *Chromobacterium violaceum* CV026 and *Agrobacterium tumefaciens* NT1 (pZLR4), showing the presence of AHLs in the crude extracts. The nature of AHLs was assessed by high-performance liquid chromatography–high-resolution tandem mass spectrometry (LC-HRMS/MS) using surface-induced dissociation (SID), as previously described [20,24]. The extracted chromatogram of the characteristic homoserine lactone product ion at *m*/*z* 102.05 allowed to trace the generating precursor AHLs, revealing the presence in the extracts of nine saturated AHL variants, along with two putative unsaturated AHLs (Table 1) [20]. Retention times and fragmentation patterns of each AHLs were compared against those of commercially available synthetic standards (details in the Experimental section and in Figure S1).

The detection of these signal molecules is often hindered by several drawbacks, mainly due to the large sample complexity. Suspecting that AHLs are produced by bacteria and not by eukaryotes, we tested whether a cell fractionation procedure that enriches the microbial fraction in the sponge, performed prior to chemical extraction, will detect AHLs in sponges. Density-gradient centrifugation-based cell fractionation was employed to enrich the microbial content among various subpopulations of cells. The first step involves the homogenization of the sponge tissue followed by sieve filtration. The first fraction (SCS-A), obtained as filtrate debris from 125 µm sieve filtration of homogenized sponge sample, containing mainly sponge cells (SCS-A C, SCS-A C:M, and SCS-A M). As expected, these sponge cells enriched fractions did not show any presence of AHLs by LC-HRMS/MS. The next five fractions (SCS-B-F), obtained by centrifugation, were expected to be enriched in microbial cells depending on differences in their density, and indeed yielded a wide variety of AHLs (Table 2), mostly the same as detected in sponge extracts (Table 1). The cell fractionation was proved to be effective, as additional seven AHLs (OHC8-AHL, OHC10-AHL, OHC12-AHL, OC12-AHL, OC14-AHL, C8:1-AHL, and OHC14:1-AHL) identified in the cell fractions were not previously detected by direct sponge tissue extraction.

Reverse-phase flash chromatography was used to create numerous fractions in order to reduce sample complexity, improve the efficiency of detection, as well as to increase the titer of minor constituents. This led to the identification of six additional long-chain AHLs (OC10-AHL, C18-AHL, OC18-AHL, C19-AHL, OC19-AHL, and C18:1-AHL) (Table 3), which were not detected when chemical analysis was performed directly on sponge crude extracts. This suggests that the previous reports on AHLs from sponges underestimated the variety of signals found in these invertebrates.

The presence of these long-chain AHLs in marine sponges may relate to their better stability at the high pH of seawater [20,40]. We report here the presence of putative C19-AHL and OC19-AHL in the AHL-enriched fraction of sponge, which, to the best of our knowledge, have never been reported from sponges. At one instance, the presence of unsaturated C19-AHL has been reported from a marine *Rhodobacteraceae* strain MOLA 401 [41]. As no standard was available, clues on the structure of the new compounds have been provided by HR-MS and HR-MS/MS spectra (Figure S2) as previously reported [20,24]. In the extracted ion chromatogram generated at *m*/*z* 102.0550, the peak at *t_R_* = 32.83 min showed a [M + H]^+^ pseudomolecular ion at *m*/*z* 382.3312, which was indicative of the molecular formula C_23_H_44_NO_3_^+^. In the HR-MS/MS spectrum, the typical fragmentation pattern of AHLs was recognized, with the homoserine lactone product ion at *m*/*z* 102.0550 and the acyl chain at *m*/*z* 281.2836, corresponding to C_19_H_37_O^+^ ion. The presence of OC19-AHL at *m*/*z* 396.3104 corresponding to the molecular formula C_23_H_41_NO_4_ was also disclosed; the coherent retention time shorter than C19 (Figure S1) and the acyl chain fragment ion at *m*/*z* 295.2632 (C_19_H_35_O_2_^+^) confirmed the hypothesis on its identity. The difference of 2 amu *m*/*z* ratio of pseudomolecular ions with the saturated AHLs and the molecular formula determination with a difference of 2 hydrogen atoms confirmed the presence of unsaturated acyl chain AHLs. Higher polarity due to unsaturation of acyl chain among unsaturated AHLs exhibited a shorter retention time when compared to its saturated counterparts (Figure S1) [20,42]. The location of the carbonyl group has only been assumed to be at position 3 due to biosynthetic origins, as for the most AHLs, but it remained unassigned [43].

### 2.3. Bioassay-guided Isolation and Structural Elucidation of ***1*** and ***2***

**Figure 2:**
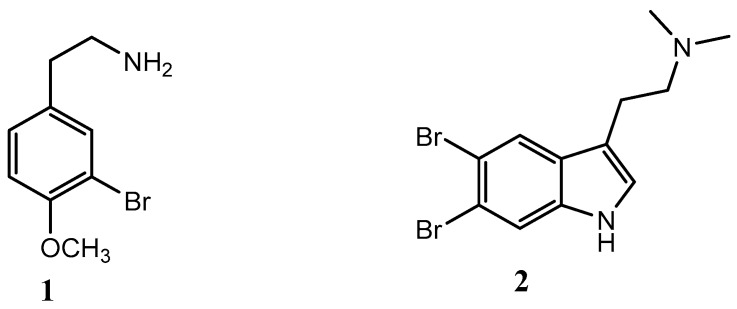
Chemical structure of compounds **1** and **2**.

The obtained crude extracts were combined and fractionated using reversed-phase flash column chromatography [44], eluting with a mixture of H_2_O/CH_3_CN (from 0 to 100%) and then with 100% of MeOH, to afford sixteen fractions (FrQ1–FrQ16). Each fraction was evaluated for potential QSI activity (see Section 3.4). Two fractions (Q1 and Q4) with potential QSI activity were further purified to obtain active molecules. Fraction Q1 was further separated by semi-preparative reversed-phase column chromatography, eluting with 10% of CH_3_CN, to obtain compound **1** (1.3 mg). Compound **2** (2.1 mg) was obtained by purification of fraction Q4 using Sephadex LH20 resulting in fifteen fractions followed by semi-preparative reversed-phase HPLC. The chemical structure of the two active molecules **1** and **2** was assessed by ^1^H NMR, as well as by HR-ESI MS. In particular, the positive ion mode HR-ESIMS of **1** and **2** displayed M+2 isotopic pseudomolecular peaks in the ratio of 1:1 and 1:2:1, respectively, accounting for the presence of one or two bromine atoms and for the molecular formula C_9_H_13_BrNO^+^ and C_12_H_15_Br_2_N_2_^+^. Taken together, MS evidence and the comparison of the ^1^H-NMR data (Figure S4), with the data reported in the literature [45,46], allowed the assignment of compounds **1** and **2** as 3-bromo-4-methoxyphenethylamine and 5,6-dibromo-*N*,*N*-dimethyltryptamine, respectively (Figure 2).

### 2.4. Determination of Non-Inhibitory Concentration (NIC)

Compounds **1** and **2** were preliminary evaluated for determination of their non-inhibitory concentration (NIC) against *E. coli* pSB401 (pSB401), *E. coli* pSB1075 (pSB1075), and *Pseudomonas aeruginosa* PAO1 (PAO1), the strains used for testing QSI activity. Determination of NIC is important to rule out the growth inhibition artifacts. The growth-inhibitory activities of compounds **1** and **2** and of the positive control, penicillic acid (PA), were tested at concentrations between 0.25 µM and 560 µM. Compounds **1** and **2** showed inhibitory activity against pSB401, pSB1075, and PAO1 only at the highest concentration, i.e., 560 µM. No growth inhibition compared with the negative control (solvent only) was observed between 0.25 µM and 280 µM. Therefore, this concentration range was used for further evaluation of QSI activity.

### 2.5. Dose-Dependent Quantification of Bioluminescence for QSI Assay

**Figure 3:**
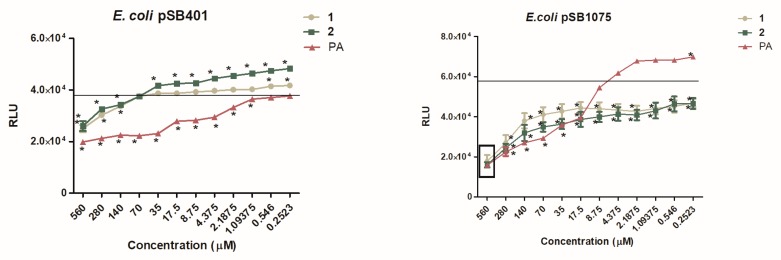
Dose-dependent effect of **1**, **2** and penicillic acid (PA) on QS-dependent bioluminescence of: A) The LuxR-based reporter *E. coli* pSB401 induced by OXO-C6-AHL; B) The LasR-based reporter *E. coli* pSB1075 induced by OXO-C12-AHL. Data are expressed as SD of mean (*n* = 3). * *P* < 0.05 versus control by ANOVA followed by Bonferroni posttest. The average bioluminescence observed for the negative control is shown by a line representing the degree of luminescence when ran without any inhibitory molecule but with its cognate AHLs.

The normalized bioluminescence results for reporter strains treated with our test compounds (**1** and **2**) and activated by incubation (4 h) with their respective cognate signal molecule are presented in Figure 3. A decrease in the bioluminescence in the presence of the test compounds **1** and **2** or the control compound PA were interpreted as QSI activity.

### 2.6. Inhibition of Production of the Virulence Factors Pyocyanin and Protease

**Figure 4:**
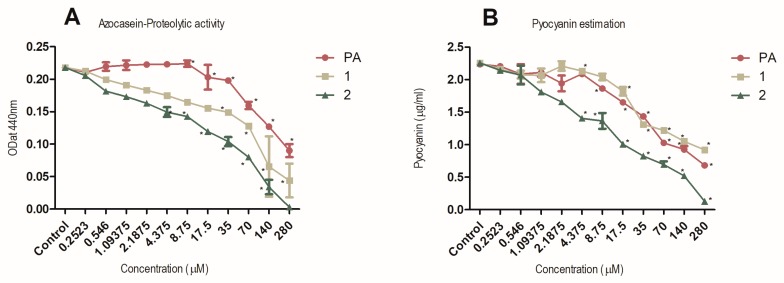
Dose-dependent inhibition of proteolytic activity (panel **A**) and pyocyanin production (panel **B**) by **1**, **2** and penicillic acid (PA). *P. aeruginosa* PAO1 grown in the presence of diluting solvent was used as negative control in both experiments. Data are expressed as SD of mean (*n* = 3). * *P* < 0.05 versus control by ANOVA followed by Bonferroni posttest.

To study the ability of compounds **1** and **2** to downregulate QS-regulated virulence factors of *P. aeruginosa* PAO1, the levels of two extracellular virulence factors were measured in the presence of the compounds. Virulence factors examined included total protease activity, which is directly controlled by the LasI/R system and pyocyanin production, which is mainly controlled by RhlI/R system. PAO1, a wild-type opportunistic pathogen strain, was used for these experiments. The activity of protease and pyocyanin production was shown to be inhibited strongly by compounds **1** and **2** in a dose-dependent manner (Figure 4).

3-Bromo-4-methoxyphenethylamine (**1**), a brominated phenethylamine, a natural monoamine alkaloid found in varieties of microbes (including fungi and bacteria), plants [47], and animal kingdoms, including humans [48], acts as a potent antimicrobial against certain pathogenic strains of *Escherichia coli* [49]. Compound **2** (5,6-dibromo-*N*,*N*-dimethyltryptamine) is a natural indole alkaloid that has also been previously isolated from the marine sponge *Hyrtios* sp. with strong antimicrobial, neurological, and antidepressant activity [45,46].

## 3. Experimental Section

### 3.1. Sponge Sampling

Five specimens of the sponge *S. spinosulus* (voucher no: 454, 455, 456, 457, and 460) were collected along the Mediterranean coast of Sdot Yam, Israel, by scuba diving at 5–12 m depth in compliance with permits n. 2012/38390 and 2013/38920 from the Israel Nature and National Parks Protection Authority. Sponges were identified morphologically following the Systema Porifera classification system [50]. Samples were placed into natural seawater using sterile scalpels and forceps and transported on ice to the laboratory for direct processing.

Each sponge sample was processed as follows; (i) Few cm from each specimen was preserved in 90% ethanol as vouchers (deposited in the Marine Microbiology Laboratory, Department Marine Biology, University of Haifa, Israel), (ii) part of each specimen was used for cell separation, and (iii) the rest of each specimen was immediately frozen in liquid nitrogen. The frozen tissues were freeze-dried using a lyophilizer, and the dried tissues were utilized for chemical extractions.

### 3.2. Taxonomic Identification of Sponge

Sponges were identified by morphological analysis and by sequencing of their COX1 genes. The primers for amplifying the mitochondrial COX1 gene were LCO1490 [51] and COX1-R1 [52]. The conditions of PCR amplifications were: 95 °C for 5 min; 35 cycles of 95 °C for 40 s, 50 °C for 50 s, 72 °C for 90 s; and a final extension at 72 °C for 10 min. The PCR products were purified using the PromegaWizard^®^ SV Gel and PCR Clean-UpSystem. COX1 amplicons with a length of 799 bp were sequenced at Macrogen Europe (1105 AZ, Amsterdam, The Netherlands) using the LCO1490 primer. For sequence alignment, additional COX1 genes sequences were downloaded from the NCBI nucleotide collection non-redundant database (http://www.ncbi.nlm.nih.gov/). The 519 bp-long final alignments were constructed. Sequences were aligned using PAGAN 0.61 [53]. The evolutionary history was inferred by using the Maximum Likelihood method based on the Hasegawa–Kishino–Yano model [54] with a discrete Gamma distribution rate variation among sites (+G). Phylogenetic robustness was inferred from 1000 bootstrap replications [55]. Evolutionary analyses were conducted in MEGA7 [56].

### 3.3. Microbial Enrichment by Cell Separation and Its Extraction

In addition to the direct extraction of sponge tissue, we also tested the potential for AHL detection in the fractions enriched with microbial cells by cell-separation. For cell separation, sponges were first washed in calcium magnesium-free seawater (CMFSW; 25 g NaCl, 0.8 g KCl, 1 g Na_2_SO_4_, 0.04 g NaHCO_3_ per 1 L) to remove loosely attached cells. The washed sponge materials were then cut into 1 cm^3^ cubes and homogenized for 10–15 s using fresh CMFSW. Microbial enrichment by cell separation was then attained using a pre-established protocol by a series of filtration and centrifugation steps as described previously [57]. Briefly, the homogenized samples were filtered through a 125 µm sieve into a sterile centrifuge tube (sample SCS-A), and the filtrate was centrifuged for 15 min at 100× *g* at 4 °C to remove remaining sponge cells and tissues (sample SCS-B). The supernatant was then centrifuged twice for 15 min at 300× *g* at 4 °C to remove the diatoms from the sample (sample SCS-C). The supernatant was afterward filtered through an 11-µm filter using the vacuum filtration unit (sample SCS-D), and the final filtrate was centrifuged for 20 min at 8800× *g* and 12,000 rpm at 4 °C to pellet microbial cells (samples SCS-E and SCS-F). The first fraction SCS-A, obtained as filtrate debris from 125 µm sieve filtration of homogenized sponge sample, was extracted with CHCl_3_ (0.5 L × 2) (SCS-A C), CHCl_3_–MeOH 1:1 (0.5 L × 2) (SCS-A C:M), and MeOH (0.5 L × 2) (SCS-A M). The next five fractions (SCS-B to F) obtained from cell separation were extracted with equal (~0.5 L) volume of butanone. The organic phase dried, resuspended in methanol, and was analyzed to check the presence of AHLs and screened for QSI compounds as described below.

### 3.4. Crude Extracts Preparation and Preliminary Screening for QSI Activity

The remaining part of all the five freeze-dried biomass specimens was macerated and repeatedly extracted with MeOH (0.5 L × 3), MeOH/CHCl_3_ (0.5 L × 2), and CHCl_3_ (0.5 L × 2) separately at room temperature. QSI activity was tested using biosensors *Chromobacterium violaceum* CV026 [58] and with an adaptation of the thin layer chromatography (TLC) overlay technique using *Agrobacterium tumefaciens* NT1 (pZLR4) [25,59]. In order to improve the detection ability of AHLs in sponge extracts, we combined all the five specimen extracts (7.8 g) and fractionated the combined extracts by reversed-phase flash column chromatography (Sigma ODS-A, 60 Å 500/400 mesh), eluting with a solvent system of 0 to 100% H_2_O/CH_3_CN and then with 100% MeOH, to afford sixteen fractions (FrS1–FrS16). The obtained fractions were then evaluated for the presence of AHLs using LC-HRMS/MS.

### 3.5. AHLs Identification Using LC-HRMS/MS Analysis

Microbial enriched crude extract fractions (SCS-A C, SCS-A C:M, and SCS-A M), crude extracts from different sponge specimens (454, 455, 456, 457, and 460), and fractions S1-S16 (obtained by fractionation of combined crude extracts from different sponge specimens) were analyzed for the presence of AHL molecules using high-resolution ESI mass spectrometry experiments (LC-HRMS and LC-HRMS/MS) using a Thermo LTQ Orbitrap XL mass spectrometer (Thermo Fisher Scientific Spa, Rodano, Italy) coupled to an Agilent model 1100 LC system (Agilent Technologies, Cernusco sul Naviglio, Italy). The spectra were recorded at positive ion mode by infusion into the ESI source using gradient elution of H_2_O and CH_3_CN both with 0.1% formic acid on a 5 μm Kinetex C18 column (50 × 2.1 mm), maintained at 25 °C, at a flow rate of 200 μL/min. The gradient program was as follows: 10% CH_3_CN for 3 min, 10%–90% CH_3_CN over 30 min, 90% CH_3_CN for 3 min. Data were collected in the Surface Induced Dissociation (SID) mode at 40 eV with a spray voltage of 5 kV, a capillary temperature of 230 °C, a sheath gas rate of 12 units N_2_ (ca. 120 mL/min), and an auxiliary gas rate of 5 units N_2_ (ca. 50 mL/min). Five microliters of a mixture of commercially available synthetic AHLs (C4-AHL, C6-AHL, OC6-AHL, C8-AHL, OC8-AHL, OHC8-AHL, C10-AHL, OC10-AHL, OHC10-AHL, C12-AHL, OC12-AHL, OHC12-AHL, C14-AHL, OC14-AHL, OHC14-AHL, C16-AHL, OC16-AHL, OHC16-AHL, C18-AHL, OC18-AHL, OHC18-AHL) were used (10 μg/mL each) to generate the extracted ion chromatogram at *m*/*z* 102.0550, corresponding to the characteristic product ion of deacylated homoserine lactone. AHLs in various fractions were identified based on the comparison of their retention time and HRMS/MS spectra with those of the synthetic standards (Figure S1) [20].

### 3.6. Bioassay-Guided Purification and Identification of Molecules with QSI Activity

The preliminary screening of sponge extracts for QSI activity (as described in the previous section) from all the five specimens displayed moderate activity, hence we collected another set of ten specimens of the same sponge species at the same location. All the specimens were combined, lyophilized (dry wt. ~121 g), macerated, and repeatedly extracted with MeOH (2 L × 3), MeOH/CHCl_3_ (1.5 L × 2), and CHCl_3_ (2 L× 2) at room temperature. The obtained crude extracts were combined and fractionated using reversed-phase flash column chromatography (Sigma ODS-A, 60 Å 500/400 mesh), eluting with a solvent system of 0 to 100% H_2_O/CH_3_CN and then with 100% of MeOH, to afford sixteen fractions (FrQ1–FrQ16). Fraction Q1 was further separated by semi-preparative reversed-phase column chromatography (Phenomenex ODS-A, 60 Å, 500/400 mesh), eluting with 10% of CH_3_CN, to obtain compound **1** (1.3 mg). Compound **2** (2.1 mg) was obtained by purification of fraction Q4 using Sephadex LH20 resulting in fifteen fractions followed by semi-preparative reversed-phase HPLC, eluting with a solvent system of 21% CH_3_CN. The NMR spectra were acquired on a Varian Unity Inova 700 MHz spectrometer equipped with a triple resonance cryoprobe (Agilent Technologies, Cernusco sul Naviglio, Italy). The chemical shifts were referenced to the residual solvent signal (CD_3_OD: δ_H_ 3.31, δ_C_ 49.01). For an accurate measurement of the coupling constants, the one-dimensional ^1^H NMR spectra were transformed at 64-K points (digital resolution: 0.09 Hz).

Compound (**1**): HRESIMS: *t_R_* = 5.0 min; [M + H]^+^ *m*/*z* 230.0167 and 232.0147 for C_9_H_13_BrNO, calcd. 230.0181 and 232.0160 (Figure S3); ^1^H NMR (700 MHz, CD_3_OD): δ 7.37 (1H, *d*, *J* = 2.2 Hz), 7.11 (1H, *dd*, *J* = 8.3, 2.2 Hz), 6.89 (1H, *d*, *J* = 8.3 Hz), 3.80 (3H, s, CH_3_-O), 2.79 (2H, *t*, *J* = 7.1 Hz), 2.63 (2H, *t*, *J* = 7.1 Hz) (Figure S4).

Compound (**2**) HRESIMS: *t*_R_ = 13.3 min; [M + H]^+^ *m*/*z* 344.9581, 346.9559 and 348.9539 for C_12_H_15_Br_2_N_2_; calcd. 344.9602, 346.9582 and 348.9561 (Figure S3); ^1^H NMR (700 MHz, CD_3_OD): 7.94 (1H, s), 7.72 (1H, s), 7.25 (1H, s), 3.22 (2H, br *t*, *J* = 7.2 Hz), 3.10 (2H, br *t*, *J* = 7.2), 2.79 (6H, s, N-(CH_3_)_2_) (Figure S4).

### 3.7. Determination of Non-Inhibitory Concentration (NIC)

The non-inhibitory concentration (NIC) was determined by the broth two-fold microdilution method (CLSI M100-S20) (CLSI 2000) for compounds **1**, **2** and PA against pSB401, pSB1075, and PAO1, the strains used for testing QSI activity. Briefly, the compounds were serially diluted (0.252–560 µM) using methanol in Muller–Hinton broth. The inoculum (approximately 5 × 10^5^ CFU/mL final concentration) was prepared from an overnight culture and was added to each well containing the compound. After incubating 96-well flat-bottomed plates aerobically at 37°C for 24 h, the optical density (OD) was measured using a spectrophotometer (600 nm) using TriStar Multimode Microplate reader (Berthold Technologies GmbH& Co. KG, Bad Wildbad, Germany) to determine NIC values. Negative controls (culture + methanol) were included. All the experiments were run in triplicates.

### 3.8. Dose-Dependent Quantification of Bioluminescence for QSI Assay

The bioluminescence-based dose-dependent QSI assay was performed using pSB401 and pSB1075 reporters and was quantified on a TriStar Multimode Microplate reader (Berthold Technologies GmbH & Co. KG, Bad Wildbad, Germany) following References [36,60]. The stock solutions (10 mM) of compounds **1**, **2** and penicillic acid (PA, positive control) were serially diluted at NIC concentrations (0.252–280 µM) for the assay. The bioluminescence was recorded every 30 min for 7 h at 30 °C. The production of bioluminescence in the graphs is given as the relative light units (RLU), obtained at 4 h [36].

### 3.9. Inhibition of Production of Virulence Factors—Pyocyanin and Protease

The inhibition of pyocyanin and protease was tested for compounds **1**, **2**, and PA (positive control) in a dose-dependent manner at NIC concentrations (0.252–280 µM) using *P. aeruginosa* PAO1 as described earlier [36]. Methanol (solvent in which test compounds were dissolved) and PA were used for negative and positive control, respectively, for both the experiments.

### 3.10. Statistical Analysis

The significant differences between the mean values of tested compounds from its corresponding controls were tested using ANOVA (*P* < 0.05) followed by Bonferroni posttest using GraphPad Prism software version 5.01. All the assays were performed in triplicates.

### 3.11. Data Deposition

Sequences of the amplified *cox1* of *S. spinosulus* were deposited in NCBI with accession numbers MK350313-MK350317.

## 4. Conclusions

In this study, we report the identification of quorum sensing activators and inhibitors in the marine sponge *Sarcotragus spinosulus*. QSI activity of two brominated alkaloids, coexisting together with QS molecules (AHLs), was showed that provides evidence for opposite functions related to cell–cell signaling within the same sponge species *S. spinosulus*. Molecules with QSI activity, such as the here identified 3-bromo-4-methoxyphenethylamine (**1**) and 5,6-dibromo-*N*,*N*-dimethyltryptamine (**2**), may be involved in a fine-tuned regulation of concentration of AHL signals through concomitant production and interference of QS signals. The potential for interaction between QS and QSI molecules within *S. spinosulus* remains to be determined. Considering the emergence due to antibiotic resistance, we really aim to work hard on this topic, together with other research groups in the world. These researches will contribute to build up the necessary knowledge to discover alternative strategies to tackle infections.

## Figures and Tables

**Table 1:** *N*-acyl homoserine lactone (AHL) variants in the crude extracts of *Sarcotragus spinosulus* (specimens 454, 455, 456, 457, and 460).

AHLs	M+H (exp)	Rt	454	455	456	457	460
OHC6:1-AHL	214.1073	1.73		x			
C6-AHL	200.1280	11.86				x	
C8-AHL	228.1593	15.39	x				
C10:1-AHL	254.1749	15.89		x		x	x
OC10-AHL	270.1697	17.46				x	
C12-AHL	284.2217	24.18					x
C14-AHL	312.2527	27.63					x
OHC14-AHL	328.2479	23.58				x	
OHC16-AHL	356.2794	25.18				x	
OHC18-AHL	384.3102	30.25					x
OC16-AHL	354.2635	25.22				x	

**Table 2:** AHL variants from the microbial enriched cell fractions of *S. spinosulus.*

AHLs	M+H (exp)	Rt	SCS-B	SCS-C	SCS-D	SCS-E	SCS-F
**C10:1-AHL**	**254.1749**	**15.89**	**x**	**x**	**x**		
**C6-AHL**	**200.1280**	**11.86**		**x**			
C8:1-AHL	226.1437	11.16	x	x			
**C8-AHL**	**228.1593**	**15.39**		**x**			
OC14-AHL	326.2320	24.96	x	x			
OHC10-AHL	272.1853	16.17		x			
OHC12-AHL	300.2168	20.08		x			
**OHC14:1-AHL**	**326.2321**	**23.86**				**x**	
OHC14-AHL	328.2479	23.58		x			
**OHC16-AHL**	**356.2794**	**25.18**			**x**		
**OHC18-AHL**	**384.3102**	**30.25**			**x**		
**OHC6:1-AHL**	**214.1073**	**1.73**		**x**			
OHC8-AHL	244.1544	10.46		x			x
OC12-AHL	298.0009	21.44		x			

Note: AHLs that were only identified by cell fractionation prior to extraction are in **bold**. Fractions (SCS-B to F) were obtained from microbial enrichment cell fractions of *S. spinosulus*.

**Table 3:** AHL variants identified from the chromatographically enriched fractions of sponge. L stands for “*low amount*”.

AHLs	M+H (exp)	rt	S1	S2	S3	S4	S5	S6
OC10-AHL	270.1697	17.44						x
OHC14:1-AHL	326.2321	23.86						x
C16-AHL	340.2845	30.71	L	x	L	L	L	
OC16-AHL	354.2635	25.22	L	x	x	L	L	
C18-AHL	368.3148	33.88	L	x	x	L	L	
OC18-AHL	382.2946	31.26	L	x	x	L	L	
OHC18-AHL	384.3103	29.95	L	x	x	L	L	
C18:1-AHL	366.2998	31.45	L	x	x	L	L	
C19-AHL	382.3312	32.83	L	x	x	L	L	
OC19-AHL	396.3104	30.20	L	x	x	L	L	

## Supplementary Materials

The following are available online at https://www.mdpi.com/1660-3397/18/2/127/s1, Figure S1: Extracted ion chromatograms at *m*/*z* 102.05, corresponding to deacylated homoserine lactone, from the LC-HRMS analysis of the microbial enriched sponge fractions: (A) standard mixture of synthetic AHLs (blue trace); (B) microbial enriched sponge fraction S2 (pink trace). Asterisk-marked AHLs (*) were only tentatively described. Figure S2: HR-MS and HR-MS/MS spectra of the new compounds C19-AHL and OC19-AHL; (a) extracted ion chromatogram from the LC-HRMS analysis of the sponge enriched fraction S2 at m/z 102.0550 (black trace), corresponding to deacylated homoserine lactone, m/z 382.3312 (pink trace), corresponding to C19-HSL and m/z 396.3104 (green trace), corresponding to OC19-AHL; (b, c) HR-MS and HR-MS/MS spectra of C19-AHL; (d, e) HR-MS and HR-MS/MS spectra of OC19-AHL. Figure S3: HR-MS spectra of compounds **1** and **2**. Figure S4: ^1^HNMR spectra of compounds **1** and **2**.
