# Supplementary material for: Identification of Quorum Sensing Activators and Inhibitors in The Marine Sponge Sarcotragus spinosulus
Source: Mar Drugs. 2020 Feb 20;18(2):127. doi: 10.3390/md18020127 (PMC7074164; doi:10.3390/md18020127)
Supplement: Supplementary file 1 [file marinedrugs-18-00127-s001.pdf]

Supplementary Information

Identification of quorum sensing activators and inhibitors in the marine sponge *Sarcotragus spinosulus*

Kumar Saurav[1,2,3], Nicola Borbone[2], Ilia Burgsdorf[1], Roberta Teta[2], Alessia Caso[2], Rinat Bar-Shalom[1], Germana Esposito[2], Maya Britstein[1], Laura Steindler[1] and Valeria Costantino[2*].

[1]Department of Marine Biology, Leon H. Charney School of Marine Sciences, University of Haifa, Mt. Carmel 31905, Haifa, Israel. sauravverma17@gmail.com (K.S); burgsdorf84@gmail.com (I.B); rbar-shal@univ.haifa.ac.il (R.B.S); mayabritstein@gmail.com (M.B); lsteindler@univ.haifa.ac.il (L.S)
[2]The Blue Chemistry Lab, Dipartimento di Farmacia, Università degli Studi di Napoli Federico II, Via D. Montesano 49, 80131, Napoli, Italy. sauravverma17@gmail.com (K.S); nicola.borbone@unina.it (N.B); roberta.teta@unina.it (R.T); alessia.caso@unina.it (A.C); germana.esposito@unina.it (G.E); valeria.costantino@unina.it (V.C)
[3]Laboratory of Algal Biotechnology-Centre Algatech, Institute of Microbiology of the Czech Academy of Sciences, Opatovický mlýn, Novohradská 237, 379 81, Třeboň, Czech Republic. sauravverma17@gmail.com (K.S)

Figure S1. Extracted ion chromatograms at *m/z*102.05, corresponding to deacylated homoserine lactone, from the LC-HRMS analysis of the microbial enriched sponge fractions.
Figure S2. HR-MS and HR-MS/MS spectra of the new compounds C19-AHL and OC19-AHL.
Figure S3. HR-MS spectra of compounds **1** and **2**.
Figure S4. ^{1}HNMR spectra of compounds **1** and **2**.

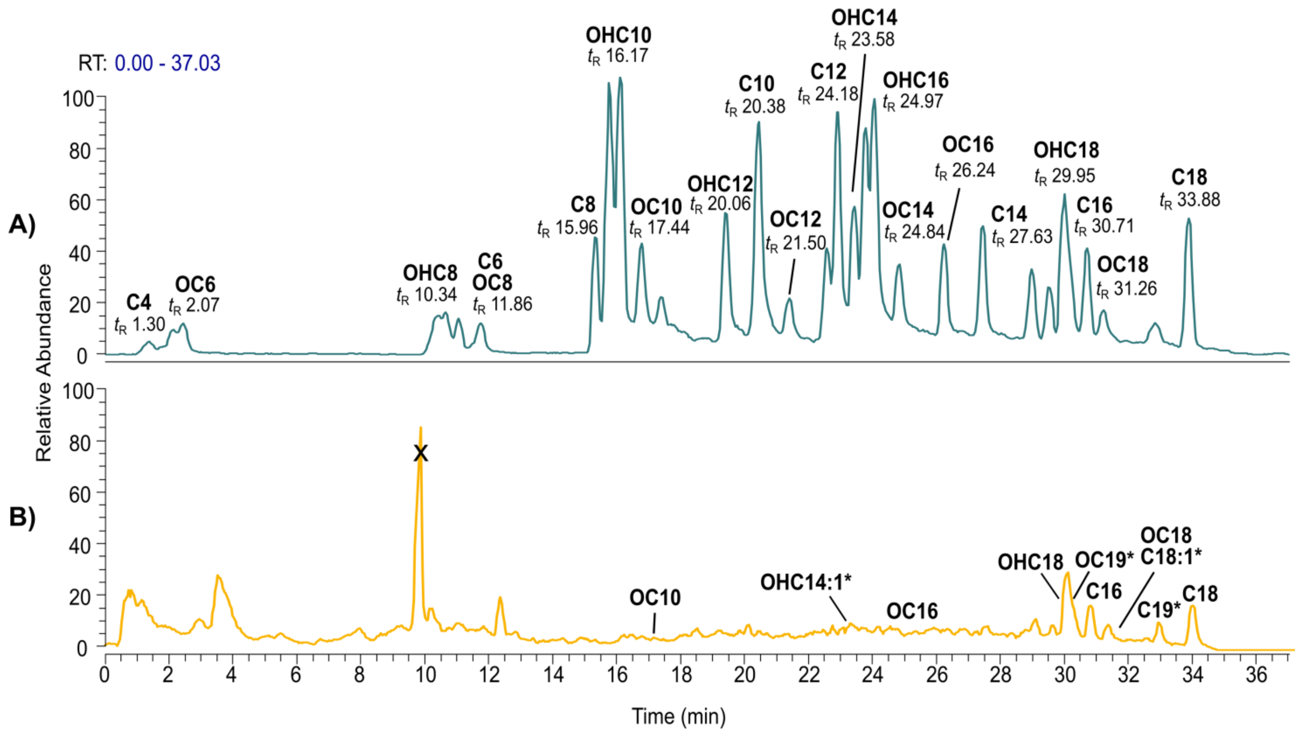

Figure S1: Extracted ion chromatograms at *m*/*z*102.05, corresponding to deacylated homoserine lactone, from the LC-HRMS analysis of the microbial enriched sponge fractions: A) standard mixture of synthetic AHLs (blue trace); B) microbial enriched sponge fraction S2 (pink trace). Asterisk-marked AHLs (*) were only tentatively described.

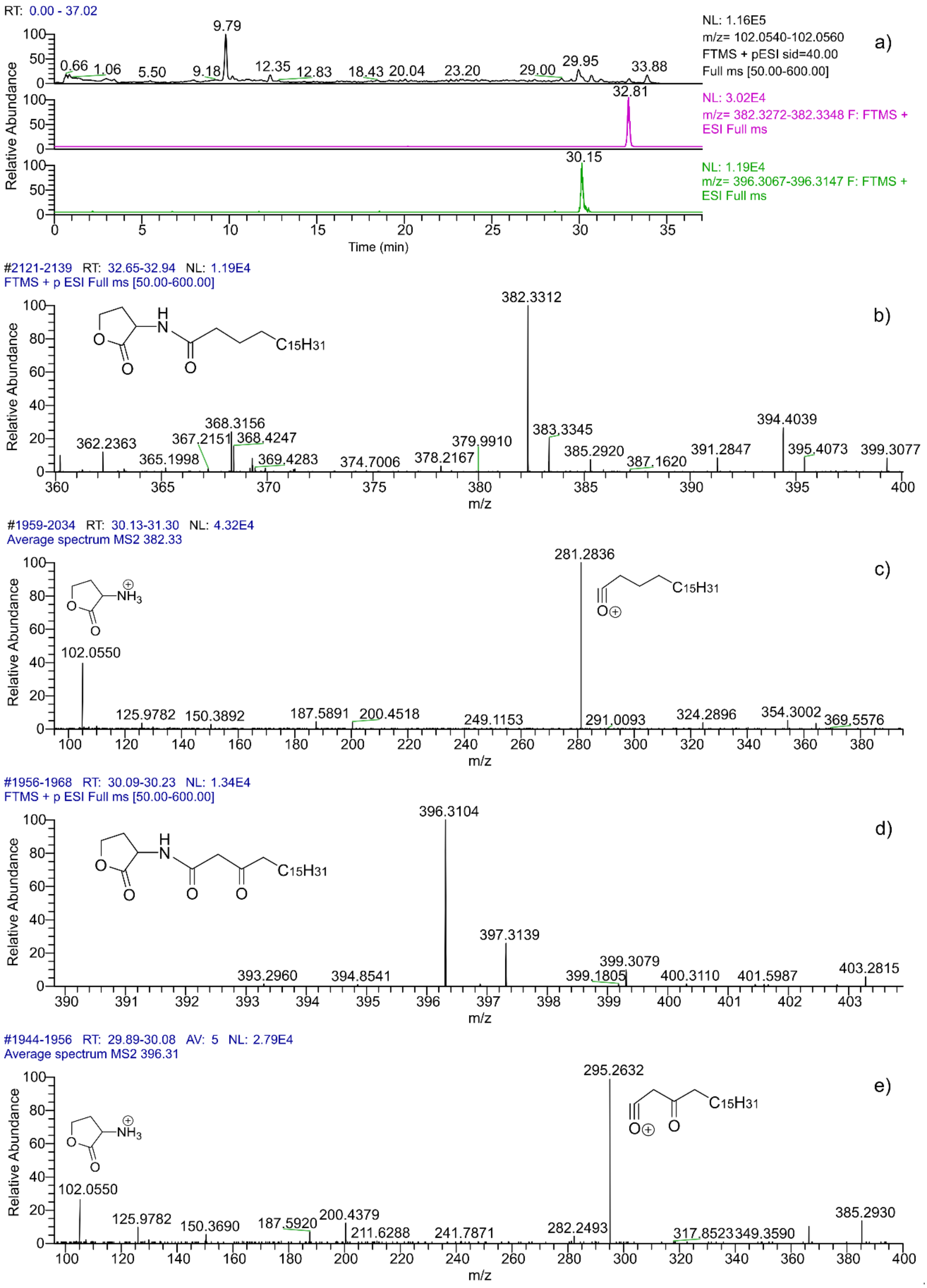

Figure S2:HR-MS and HR-MS/MS spectra of the new compounds C19-AHL and OC19-AHL; a) extracted ion chromatogram from the LC-HRMS analysis of the sponge enriched fraction S2 at *m/z* 102.0550 (black trace), corresponding to deacylated homoserine lactone, *m/z* 382.3312 (pink trace), corresponding to C19-AHL and *m/z* 396.3104 (green trace), corresponding to OC19-AHL; b), c) HR-MS and HR-MS/MS spectra of C19-AHL; d), e) HR-MS and HR-MS/MS spectra of OC19-AHL.

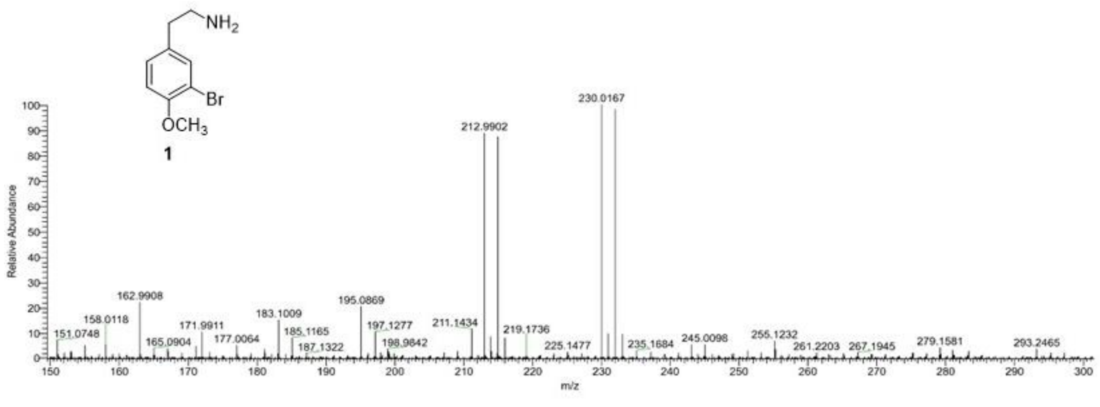

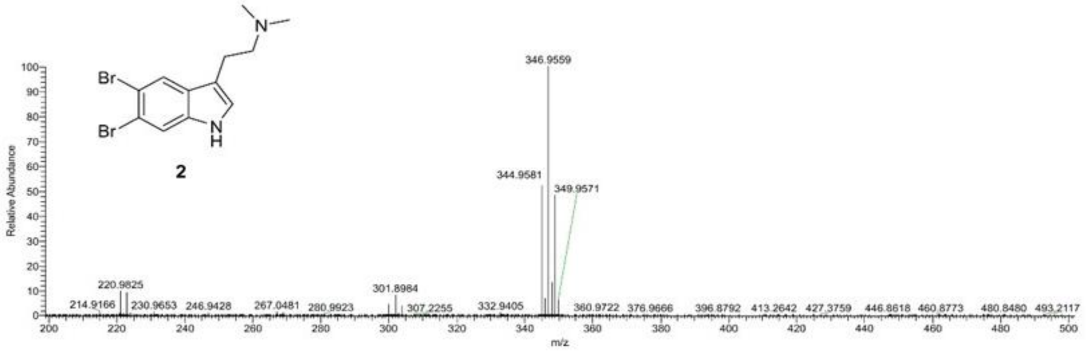

Figure S3:HR-MS spectra of compounds **1** and **2**.

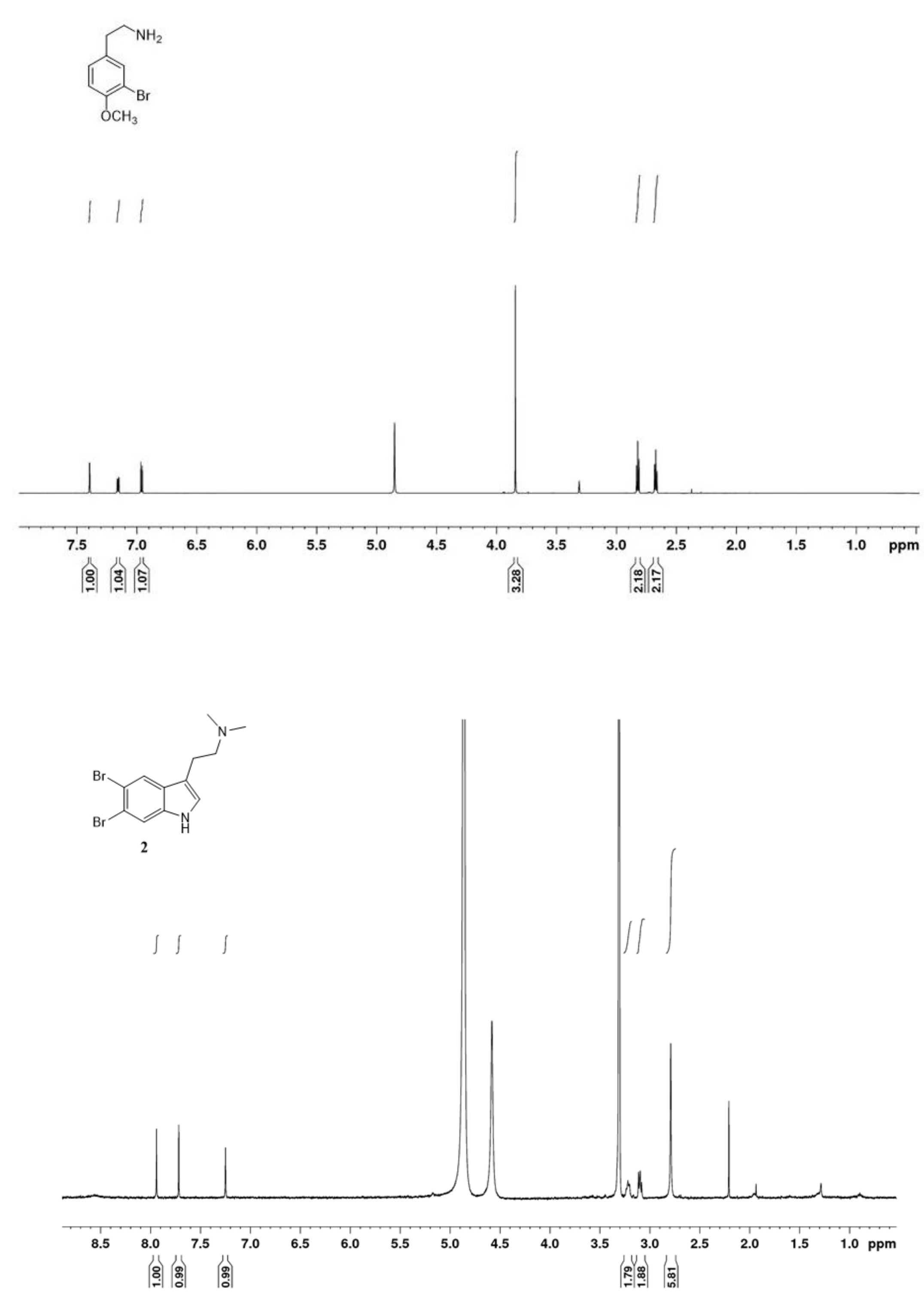

Figure S4: ^{1}HNMR spectra of compounds **1** and **2**.
